# Argonaute 2 Restores Erectile Function by Enhancing Angiogenesis and Reducing Reactive Oxygen Species Production in Streptozotocin (STZ)-Induced Type-1 Diabetic Mice

**DOI:** 10.3390/ijms24032935

**Published:** 2023-02-02

**Authors:** Fang-Yuan Liu, Guo Nan Yin, Jiyeon Ock, Fitri Rahma Fridayana, Lashkari Niloofar, Yan Huang, Minh Nhat Vo, Jun-Kyu Suh, Soon-Sun Hong, Ju-Hee Kang, Ji-Kan Ryu

**Affiliations:** 1 National Research Center for Sexual Medicine and Department of Urology, Inha University College of Medicine, Incheon 22332, Republic of Korea; 2 Department of Biomedical Sciences, College of Medicine and Program in Biomedical Science & Engineering, Inha University, Incheon 22332, Republic of Korea; 3 Department of Pharmacology and Medicinal Toxicology Research Center, Inha University College of Medicine, Incheon 22332, Republic of Korea

**Keywords:** Ago2, angiogenesis, diabetes, erectile dysfunction, reactive oxygen species

## Abstract

Severe vascular and nerve damage from diabetes is a leading cause of erectile dysfunction (ED) and poor response to oral phosphodiesterase 5 inhibitors. Argonaute 2 (Ago2), a catalytic engine in mammalian RNA interference, is involved in neurovascular regeneration under inflammatory conditions. In the present study, we report that Ago2 administration can effectively improve penile erection by enhancing cavernous endothelial cell angiogenesis and survival under diabetic conditions. We found that although Ago2 is highly expressed around blood vessels and nerves, it is significantly reduced in the penis tissue of diabetic mice. Exogenous administration of the Ago2 protein restored erectile function in diabetic mice by reducing reactive oxygen species production-signaling pathways (inducing eNOS Ser^1177^/NF-κB Ser^536^ signaling) and improving cavernous endothelial angiogenesis, migration, and cell survival. Our study provides new evidence that Ago2 mediation may be a promising therapeutic strategy and a new approach for diabetic ED treatment.

## 1. Introduction

Diabetes mellitus (DM), a primary cause of erectile dysfunction (ED), is a pancreatic β-cell dysfunction that leads to insulin deficiency and resistance, resulting in microvascular complications [1]. Epidemiological surveys have shown that approximately 75% of men with diabetes also suffer from ED, which is caused by severe subsequent damage to cavernous microvasculature and nerves [2]. Furthermore, men with diabetic ED are less responsive to oral phosphodiesterase 5 inhibitors than men without diabetes [3,4,5]. Researchers have developed many new methods and targets for this phenomenon, such as therapies regarding stem cells, low-intensity extracorporeal shockwaves, and angiogenic and neurotrophic factors [6,7,8,9,10,11]. However, due to the various side effects and different etiologies, clinical application is not ideal, making it necessary to explore new therapeutic avenues.

Argonaute 2 (Ago2) is essential for mammalian development as it is a catalytic engine of mammalian RNA interference [12]. Recent studies of Ago2–miRNA complexes in human plasma suggest that Ago2 may play an important role in the stability of secreted miRNA [13]. Of the four Ago proteins found in humans, only Ago2 is present in mitochondria, can directly interact with mitochondria ribosomal RNA, and is associated with other mitochondrial proteins involved in mitochondrial ribosome assembly and translation. The discovery of miRNA and Ago2 within mitochondria presents a potential new mode of regulation of mitochondrial DNA-encoded genes for the regulation of oxidative phosphorylation [14,15,16]. In addition, Ago2 is involved in angiogenesis through the vascular endothelial growth factor signaling pathway [17] and may be considered a putative therapeutic target in the neurovascular unit under inflammatory conditions [18]. Exogenous application of Ago2 may increase intracellular levels through Neuropilin-1 (NRP1)-mediated internalization. As the main Ago2 receptor, NRP1 is mainly expressed in neuronal, endothelial, and epithelial cells, thus responding to extracellular Ago2 [18]. Systemic administration of Ago2 restores endothelium, normalizes microglia response in vitro, and normalizes endothelial and glial activation in the mouse cortex in vivo [18]. Ago2 has also been proven to be related to pancreatic β-cell compensatory expansion [19], cell differentiation [20], and skeletal muscle homeostasis [21]. Nuclear Ago2 regulates adipose tissue-derived stem cell survival by controlling reactive oxygen species (ROS) levels [22]. More recently, Barend W. et al. elucidated that Ago2 may play an important role in restoring microvascular damage in diabetic nephropathy by inducing endothelial barrier formation and angiogenesis [23]. However, the detailed mechanisms of Ago2 in diabetic ED are still poorly understood.

In the present study, we aimed to determine whether an exogenous injection of Ago2 proteins can improve erectile function in streptozotocin (STZ)-induced type-1 diabetic mice and explore the specific mechanisms of Ago2 in this process. As Ago2 was reduced in the penis of diabetic mice, we found that intracavernous delivery of Ago2 proteins decreased ROS levels and induced the survival of cavernous endothelial and neuronal cells, effectively improving erectile function. These results were also validated in human umbilical vein endothelial cells in vitro under high-glucose conditions.

## 2. Results

### 2.1. Physiologic and Metabolic Parameters

Compared with age-matched control mice, body weight was significantly lower in STZ-induced type-1 diabetic mice, but fasting and postprandial blood glucose concentrations were significantly higher. However, these parameters did not significantly change, regardless of treatment. No detectable difference in MSBP was found across all experimental groups (Table S1).

### 2.2. Ago2 Expression Decreases in Diabetic and High-Glucose Conditions

**Figure 1:**
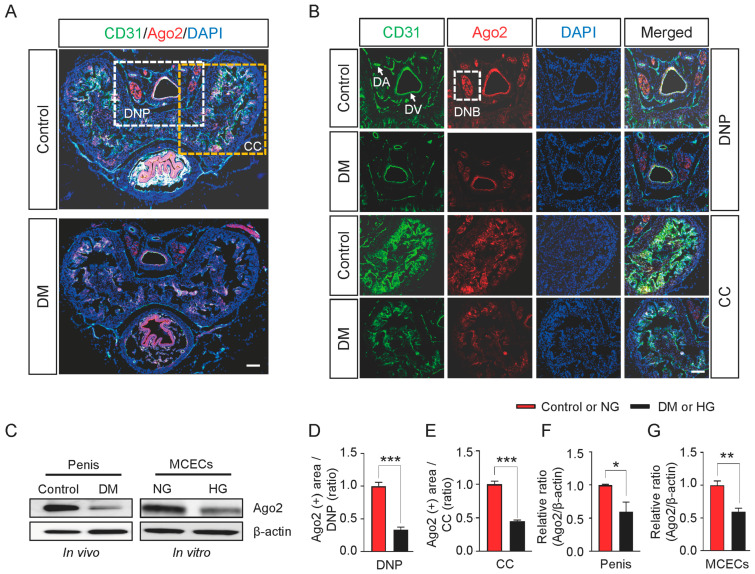
Ago2 expression under normal and diabetic conditions. (**A**) Representative images of immunofluorescent stains for cavernosum tissues of age-matched control and diabetic mice against CD31 (green) and Ago2 (red) antibodies. Scale bar: 200 μm. Nuclei were labeled with DAPI (blue). (**B**) Higher-magnification images of CD31 (green) and Ago2 (red) in the DNP (**top panel**) and CC (**bottom panel**). Scale bar: 100 μm. (**C**) Representative Western blots for Ago2 in age-matched control and diabetic mice penis tissue in vivo and MCECs exposed to NG or HG conditions in vitro. (**D**–**G**) Ago2-immunopositive areas in DNP (**D**), CC (**E**), and relative Western blot band intensity values (**F**,**G**) were quantified using ImageJ software (n = 4, * *p* < 0.05, ** *p* < 0.01, *** *p* < 0.001). The results were presented as means ± SEMs. The value expressed as ratios of the control or NG group was arbitrarily set to 1. DM, diabetes mellitus; DNP, dorsal nerve part; CC, corpus cavernosum; DA, dorsal artery; DV, dorsal vein; DNB, dorsal nerve bundle; DAPI, 4,6-diamidino-2-phenylindole; MCECs, mouse cavernous endothelial cells; NG, normal glucose; and HG, high glucose.

Double immunostaining for CD31 (an endothelial cell marker, green) and Ago2 (red) was utilized to comprehensively characterize the distribution and expression of Ago2 in the mouse penis tissue. This revealed that Aog2 is highly colocalized with CD31 expressed in the corpus cavernosum (CC) and dorsal nerve part (DNP) of mouse penis tissue (Figure 1A,B). We also noted a significantly higher expression in the dorsal vein (DV) and less in the dorsal artery (DA), as indicated by the arrows (Figure 1B). Interestingly, Aog2 also maintained a high expression in the dorsal nerve bundle (DNB), illustrated by the white dashed box (Figure 1B). Next, we found that the Ago2 expression significantly decreased in the penis tissues of STZ-induced type-1 diabetic mice (DM) compared with age-matched controls (Figure 1A,B,D,E). Western blot analyses also showed that Ago2 expression significantly decreased in diabetic conditions in vivo (penis tissues, Figure 1C, left and Figure 1F) and high-glucose conditions in vitro (MCECs, Figure 1C, right, and Figure 1G). These results indicate that Ago2 expression is high in neurovascular tissues and low under diabetic conditions in vivo and in vitro, which may be related to neurovascular regeneration.

### 2.3. Ago2 Improves Angiogenesis under High-Glucose Conditions

**Figure 2:**
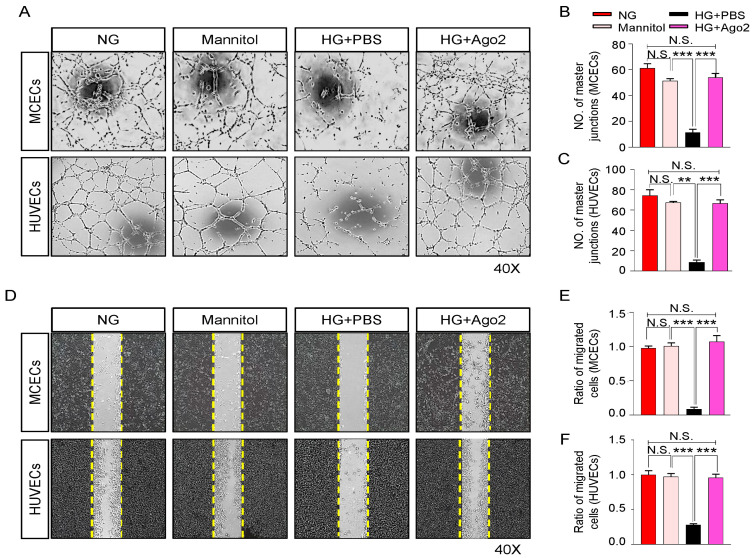
Ago2 induces tube formation and migration of endothelial cells under high-glucose conditions. (**A**–**C**) Tube formation assays in MCECs (**A**, **top**) and HUVECs (**A**, **bottom**) exposed to NG, mannitol (an osmotic control), HG with PBS, or Ago2 proteins (200 ng/mL). (**B**,**C**) The number of master junctions in MCECs (**B**) and HUVECs (**C**) were quantified using ImageJ software (n = 4, ** *p* < 0.01, *** *p* < 0.001). (**D**–**F**) Migration assay in MCECs (**D**, **top**) and HUVECs (**D**, **bottom**) with the same conditions stated above. The number of migrated MCECs (**E**) and HUVECs (**F**) in the frame line was quantified using ImageJ software (n = 4, *** *p* < 0.001). Magnification, 40×. The results were presented as means ± SEMs. The value expressed as ratios of the NG group was arbitrarily set to 1. MCECs, mouse cavernous endothelial cells; HUVECs, human umbilical vein endothelial cells; NG, normal glucose; HG, high glucose; and N.S., not significant.

It is well-known that Ago2 mediates angiogenesis by targeting angiogenesis-related genes [24]. To assess whether Ago2 influences angiogenesis, we performed tube formation and migration arrays in MCECs and HUVECs. We found that well-organized capillary-like structures (Figure 2A–C and Figure S1) and a number of migrated cells (Figure 2D–F) significantly decreased under PBS-treated high-glucose conditions compared with normal-glucose or mannitol-treated (an osmotic control) conditions. However, Ago2 treatment significantly ameliorated these effects under high-glucose conditions (Figure 2). These results validate the angiogenic role of Ago2 in endothelial cells.

### 2.4. Exogenous Injection of Ago2 Improves Erectile Function in Diabetic Mice

**Figure 3:**
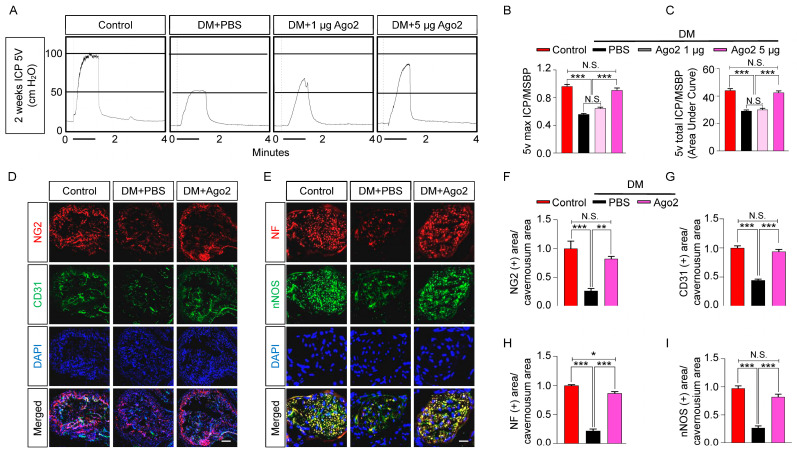
Ago2 improves erectile function in diabetic mice. (**A**) Representative ICP responses in age-matched control and diabetic mice 2 weeks after two intracavernous injections (days −3 and 0) of phosphate-buffered saline (PBS, 20 μL) or Ago2 proteins (1, 5 μg in 20 μL of PBS, respectively). The solid bar indicates the stimulus interval. (**B**,**C**) Ratios of maximum ICP (**B**) and total ICP (**C**) to MSBP were calculated for each group (n = 10, *** *p* < 0.001). (**D**,**E**) Representative images of immunofluorescent stains of corpus cavernosum and dorsal nerve bundle tissues from age-matched control and diabetic mice 2 weeks after two intracavernous injections (days −3 and 0) of phosphate-buffered saline (PBS, 20 μL) or Ago2 proteins (5 μg in 20 μL of PBS) for NG2 (red), CD31 (green), NF (red), and nNOS (green) after ICP studies. Nuclei were labeled with DAPI (blue). Scale bars: 100 µm (**D**) and 25 µm (**E**). (**F**–**I**) Quantitative analysis of corpus cavernosum pericyte ((**F**), NG2), endothelial cell ((**G**), CD31), and neuronal cell ((**H**), NF; (**I**), nNOS) contents using ImageJ software (n = 4, *** *p*< 0.001). The results were presented as means ± SEMs. The value expressed as ratios of the control group was arbitrarily set to 1. DM, diabetes mellitus; ICP, intracavernous pressure; MSBP, mean systolic blood pressure; NF, neurofilament; DAPI, 4,6-diamidino-2-phenylindole; and N.S., not significant.

To explore the role of Ago2 in neurovascular regeneration, we assessed the intracavernous pressure (ICP) by electrically stimulating the cavernous nerve (5 V, 12 Hz, and 1 m/s) 2 weeks after intracavernous injection of PBS or different doses of Ago2 (days −3 and 0; 1, 5, and 20 µg in 20 μL of PBS) in age-matched control and diabetic mice (Figure 3A). Compared with the age-matched control mice, the ratios of maximum and total ICP to mean systolic blood pressure (MSBP) were significantly lower in the PBS-treated diabetic mice. We found erection parameters significantly improved in the 5 µg of Ago2 treatment group (achieved 90% of control values). However, recovery of erectile function was not evident in the 1 µg Ago2 treated group. In addition, we could not obtain function results in the Ago2-treated group at high doses (20 μg/20 μL of PBS) due to severe cavernous tissue inflammation (Figure 3A–C). Immunofluorescent staining of cavernous tissue with CD31 (an endothelial cell marker), NG2 (a pericyte marker), neurofilament (NF, a neuronal cell marker), and neuronal nitric oxide synthase (nNOS, a neuronal cell marker) antibodies revealed that the Ago2 protein significantly improved pericyte (Figure 3D,F), endothelial cell (Figure 3D,G), and neuronal cell (Figure 3E,H for NF and Figure 3E,I for nNOS) content in diabetic mice. Altogether, these results indicate that Ago2 promotes the cavernous endothelial mural and neuronal cell content, thereby improving erectile function in diabetic mice.

### 2.5. Ago2 Induces Endothelial Proliferation and Reduces Apoptosis under Diabetic Conditions In Vivo and In Vitro

**Figure 4:**
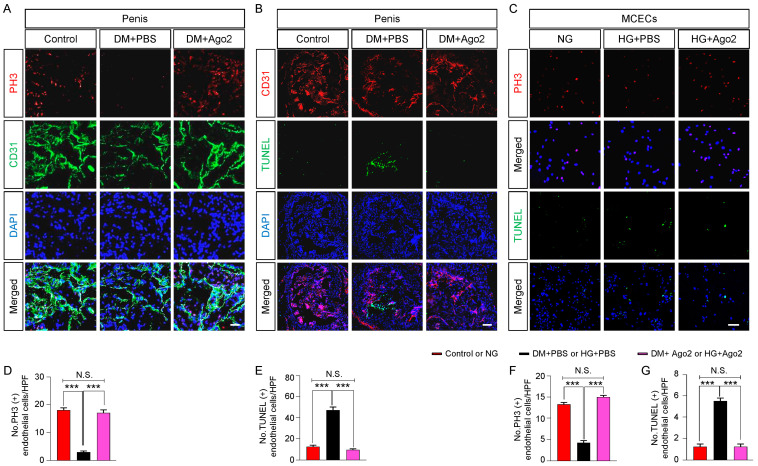
Ago2 induces endothelial proliferation and reduces apoptosis in diabetic conditions in vivo and in vitro. (**A**) Immunofluorescence staining of corpus cavernosum tissue for phospho-histone H3 (PH3; red) and CD31 (green) in age-matched control and diabetic mice 2 weeks after two intracavernous injections (days −3 and 0) of phosphate-buffered saline (PBS, 20 μL) or Ago2 proteins (5 μg in 20 μL of PBS). Scale bars: 100 µm. (**B**) TUNEL assay (green) and CD31 (red) immunofluorescence staining of corpus cavernosum tissue using the same samples above. Scale bars: 100 µm. (**C**) Immunofluorescence staining for PH3 (red, **C**, **top panel**) and TUNEL (green, **C**, **bottom panel**) in MCECs exposed to NG, or HG with PBS or Ago2 proteins (200 ng/mL), respectively. Scale bars: 50 µm. Nuclei were labeled with DAPI (blue). (**D**–**G**) The number of PH3-positive or TUNEL-positive endothelial cells in vivo (**D**,**E**) and in vitro (**F**,**G**) were quantified using ImageJ software (n = 4, *** *p* < 0.001). The results were presented as means ± SEMs. DM, diabetes mellitus; TUNEL, terminal deoxynucleotidyl transferase-mediated deoxyuridine triphosphate nick end labeling; DAPI, 4,6-diamidino-2-phenylindole; MCECs, mouse cavernous endothelial cells; NG, normal glucose; HG, high glucose; and N.S., not significant.

Diabetic endothelial dysfunction faces a process of reduced proliferation and increased apoptosis in diabetes, thereby affecting angiogenesis. Overexpression of Ago2 promotes proliferation in hepatocellular carcinoma [25], while Ago2 knock-down induces apoptosis in myeloid leukemia cells [26]. Through immunofluorescent staining, we examined cavernous endothelial cell proliferation (PH3, Figure 4A,C,D,F) and apoptosis (TUNEL assay, Figure 4B,E,C,G) in cavernous tissues and MCECs treated with Ago2 under diabetic or high-glucose conditions. We found severely reduced proliferation and increased apoptosis under diabetic conditions in vivo (diabetic tissues, Figure 4A,B,D,E) and in vitro (MCECs under high-glucose conditions, Figure 4C,F,G). However, these effects were restored to normal values after Ago2 protein treatment (Figure 4). The same effect was also verified in HUVECs exposed to high-glucose conditions (Figure S2). Collectively, these data suggest that Ago2 induces cavernous endothelial cell survival by increasing proliferation and decreasing apoptosis under diabetic conditions. 

### 2.6. Ago2 Reduces Reactive Oxygen Species in Cavernous Tissue of Diabetic Mice

**Figure 5:**
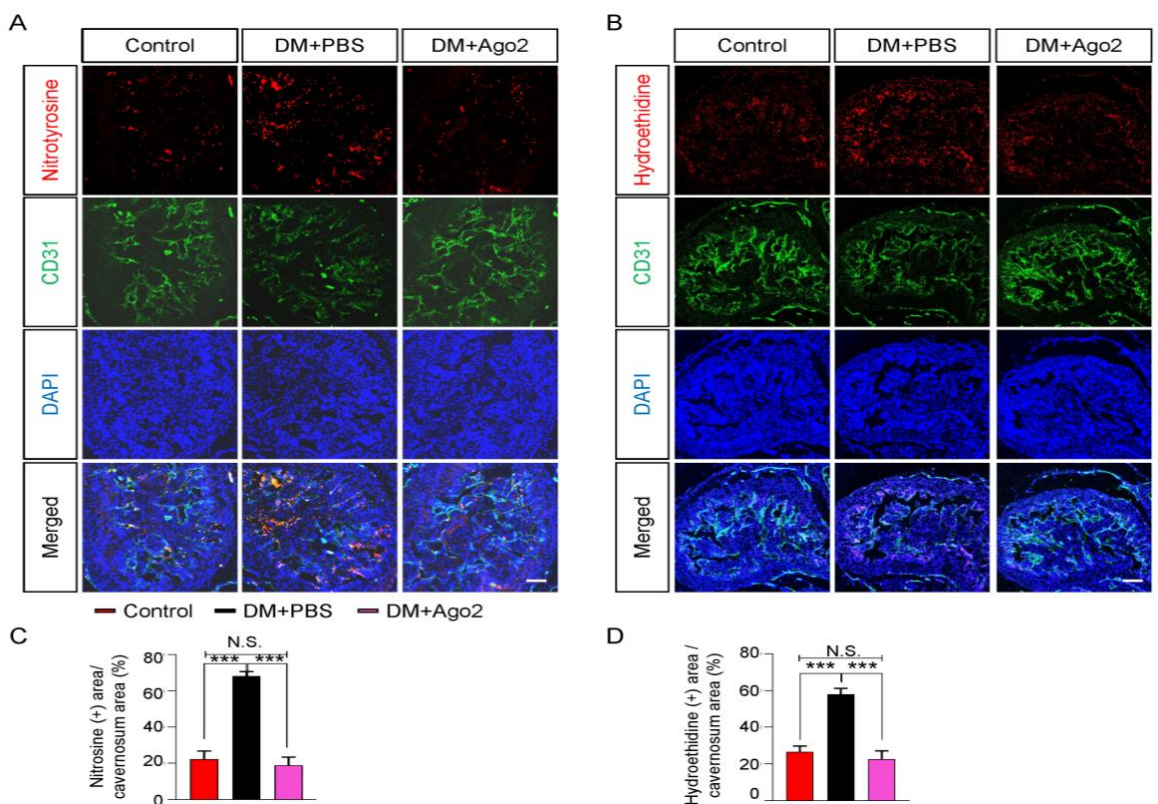
Ago2 reduces ROS production in diabetic mice. (**A**) Immunofluorescence double staining of corpus cavernosum tissue for nitrotyrosine (a marker of peroxynitrite generation, red) and CD31 (green) in age-matched control and diabetic mice 2 weeks after two intracavernous injections (days −3 and 0) of phosphate-buffered saline (PBS, 20 μL) or Ago2 proteins (5 μg in 20 μL of PBS). Scale bars: 100 µm. (**B**) In situ detection of hydroethidine (an oxidative fluorescent dye used to detect superoxide anions, red) production and CD31 (green) in corpus cavernosum tissue by using the same samples above. Scale bars: 100 µm. Nuclei were labeled with DAPI (blue). (**C**,**D**) The nitrotyrosine-immunopositive cavernosum area (**C**) and the hydroethidine-immunopositive cavernosum area (**D**) were quantified using ImageJ software (n = 4, *** *p* < 0.001). The results were presented as means ± SEMs. DM, diabetes mellitus; DAPI, 4,6-diamidino-2-phenylindole; ROS, reactive oxygen species; and N.S., not significant.

Diabetes-induced production of reactive oxygen species (ROS) is associated with oxidative stress, inflammation, and cellular death [27]. Therefore, we performed immunofluorescence staining for nitrotyrosine and hydroethidine to detect peroxynitrite and superoxide anion production in the cavernous tissues of diabetic mice. We found that nitrotyrosine and oxidized hydroethidine expression in cavernous tissues was significantly higher in the PBS-treated diabetic mice. However, an intracavernous Ago2 injection significantly lowered peroxynitrite (Figure 5A,C) and superoxide anion (Figure 5B,D) production. These results suggest that Ago2 can reduce ROS damage to endothelial cells in diabetic mice.

### 2.7. Ago2 Induces Cavernous eNOS Ser^1177^ and NF-κB Ser^536^ Phosphorylation under Diabetic Conditions Mouse Penis Tissues and MCECs

**Figure 6:**
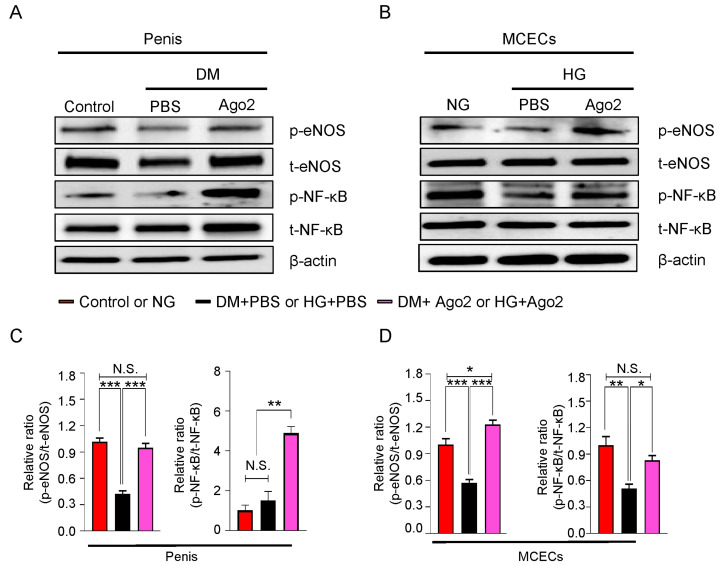
Ago2 induces eNOS Ser^1177^ and NF-κB Ser^536^ phosphorylation in diabetic conditions in vivo and in vitro. (**A**) Representative Western blots for p-eNOS Ser^1177^, total eNOS, p-NF-κB Ser^536^, and total NF-κB in corpus cavernosum tissue from age-matched control and diabetic mice at 2 weeks after two intracavernous injections (days −3 and 0) of phosphate-buffered saline (PBS, 20 μL) or Ago2 proteins (5 μg in 20 μL of PBS). (**B**) Representative Western blots for the same targets above in MCECs exposed to NG or HG with PBS or Ago2 proteins (200 ng/mL). (**C**,**D**) Normalized band intensity values for indicated targets in the penis (**C**) and MCECs (**D**) were quantified using ImageJ software (n = 4, * *p* < 0.05, ** *p* < 0.01, and *** *p* < 0.001). The results were presented as means ± SEMs. The value expressed as ratios of the control group was arbitrarily set to 1. DM, diabetes mellitus; MCECs, mouse cavernous endothelial cells; NG, normal glucose; HG, high glucose; and N.S., not significant.

Ago2 administration normalizes endothelial cells in the mouse cortex and exerts angiogenic effects in human hepatocellular carcinoma through VEGF signaling [17,18]. To explore this mechanism, we evaluated the expression of p-eNOS Ser^1177^/total-eNOS (t-eNOS) and p-NF-κB Ser^536^/total-NF-κB (t-NF-κB) in mouse cavernous tissues and MCECs under diabetic conditions. eNOS Ser^1177^ phosphorylation was significantly decreased under diabetic conditions but was restored in Ago2-protein-treated mouse penis tissues (Figure 6A,C, left), MCECs (Figure 6B,D, left), and HUVECs (Figure S3A,B, left). In addition, even NF-κB Ser^536^ phosphorylation was significantly decreased only within in vitro conditions (but not in vivo conditions), NF-κB Ser^536^ phosphorylation was significantly increased in Ago2-protein-treated penis tissues (Figure 6A,C, right), and MCECs (Figure 6B,D, right) under diabetic conditions, but not HUVECs (Figure S3A,B, right). These results suggest that Ago2 promotes angiogenesis under diabetic conditions by promoting eNOS and NF-κB signaling pathways of endothelial cells, ultimately ameliorating erectile dysfunction in diabetic mice.

## 3. Discussion

Argonaute proteins have been implicated in RNA silencing and related phenomena in several organisms [28]. They contain two common domains, PAZ and PIWI [29]. PAZ is a protein–protein interaction domain [29], including four functional Argonaute proteins (Ago1–4) that share high structural similarities in humans and mice [30]. Compared with other Agos, only Ago2 plays an important role in the early development of humans and mice, determining cell fate [28,30]. The present study shows that Ago2 is highly expressed in the neurovascular part of cavernous tissue and is significantly decreased under pathological conditions, such as diabetes. Therefore, we hypothesized that the decreased expression of Ago2 might be involved in the vasculopathy phenomena in diabetic ED.

To test our hypothesis, we performed tube formation and migration arrays using MCECs and HUVECs under HG conditions to mimic diabetes-induced angiopathy. We found that treatment with mouse Ago2 recombinant proteins significantly improved the reduced capillary-like structures and the migratory ability of MCECs in a dose-dependent manner under HG conditions (Figure S1). Therefore, by evaluating the effects of different Ago2 protein doses on angiogenesis and cell migration under HG conditions, we chose 200 ng/mL as the in vitro treatment dose. Although Ago2 is a highly conserved protein with about 99.1% amino acid identity between humans and mice [31], the effect of mouse recombinant Ago2 in NF-κB Ser^536^ phosphorylation was not evident in HUVECs. This may be because the signaling response time of NF-κB Ser^536^ phosphorylation may be earlier and faster, or the protein is s recombinant mouse form; these need further studies for proof.

We used a diabetes-induced ED mouse model to evaluate the angiogenic effect of Ago2 and its optimal therapeutic dose within in vivo studies. We chose this model because the destruction of pancreatic β-cells by STZ can lead to a phenotype similar to insulin-dependent type 1 diabetes [32]. This model has been utilized in many studies regarding microvascular complications caused by type 1 diabetes [33,34,35,36,37,38]. Our results showed that, from diabetic ED mice treated with three doses of Ago2 (1, 5, and 20 µg), 5 µg was the optimal therapeutic dose because of its superior erection-recovery parameters, reaching a 90% value of the control. However, this study only evaluated the recovery of erectile function after 2 weeks of Ago2 treatment, and due to the longevity and high price of the recombinant protein, we could not evaluate the effect of Ago2 treatment for a longer period of time. In further studies, the gene encoding Ago2, such as lentiviral particles, will address this deficiency. In the present study, we found that the Ago2 protein treatment significantly improved pericyte, endothelial cell, and neuronal cell (Figure 3) survival in diabetic mice. These cells contribute to maintaining the expression of cell junction proteins and rescue vascular and nerve abnormalities in cavernosal tissue. In addition, Machado-Pereira M. et al. demonstrated that Ago2 treatment preserves tight junction protein levels under lipopolysaccharide-induced inflammatory conditions [18]. Several studies have shown that the cell–cell communication via the gap and tight junctions is important in the pathogenesis of diabetes conductance in different cells, such as pericytes and endothelial cells [39,40,41,42]. Therefore, we believe that further studies are needed to demonstrate whether Ago2 can also restore and maintain erectile function by regulating the expression of cell junction proteins (especially in cavernous pericyte and endothelial cells) in diabetic conditions. Altogether, our results confirmed the hypothesis that Ago2 plays an important role in restoring erectile function by inducing angiogenesis in diabetic ED.

Previous studies have shown that the overproduction of reactive oxygen species (ROS), such as superoxide anions and peroxynitrite, is the main cause of eNOS inactivation in the corpus cavernosum of diabetic mice [43,44]. In diabetic conditions, high levels of ROS can reduce cell proliferation and induce cell apoptosis [45,46,47]. Interestingly, Ago2 is also known to control ROS levels and exert its cell survival role against ROS-induced cell death [22]. In the present study, we found that the Ago2 treatment group restored cavernous endothelial content by increasing endothelial cell proliferation and reducing apoptosis, reducing ROS production in diabetic mice overall. Therefore, the reduction of ROS production may be one main mechanism of Ago2-mediated improvement of cavernous neurovascular integrity and, ultimately, erectile function. Ago2 increased phosphorylation in eNOS Ser^1177^ and NF-κB Ser^536^ under diabetic conditions in both in vivo and in vitro trials, even though these results were not all validated in HUVECs. NF-κB is a key regulator of innate and adaptive immune responses, can stimulate inflammatory processes, and has recently been implicated in multiple aspects of angiogenesis [48,49,50]. Therefore, it is necessary to identify further NF-κB target genes related to Ago2-mediated angiogenesis and nerve regeneration. In sum, our results suggest that Ago2 treatment can improve erectile function by modulating ROS-mediated cell death in diabetic mice. However, the current study did not explain the detailed mechanism between Aog2 and ROS. Based on the known literature [16,51,52], we speculate that Ago2 present in mitochondria may regulate the imbalance between mitochondrial reactive oxygen species (mtROS) production and clearance by regulating ROS overproduction and/or reducing antioxidant defense activity by restoring mitochondrial function and eventually restoring erectile function. This requires further studies to assess whether Ago2 has a direct or indirect effect on the functional recovery of mitochondria in corpus cavernosum cells.

To the best of our knowledge, this study is the first to demonstrate the efficacy of Ago2 in improving erectile function in diabetes-induced ED mice. Nevertheless, our study has a few limitations. First, although we demonstrated that Ago2 can ameliorate endothelial dysfunction under diabetic conditions, we did not assess whether Ago2 also exerts a similar effect in other cell types, such as pericyte or neuronal cells. Second, the present study only referred to the known roles of Ago2 and did not conduct a more in-depth analysis of Ago2′s action mechanism, such as whether Ago2 can also promote neurovascular regeneration by regulating other signaling pathways or miRNAs. This requires further research into global gene expression analysis using next-generation sequencing-based technologies, such as single-cell sequencing analysis. Third, we only described changes in fasting and postprandial blood glucose levels; therefore, we cannot postulate the development of diabetes, and it is necessary to perform glucose and insulin tolerance tests in future studies using STZ-induced type-1 diabetic mice.

## 4. Materials and Methods

### 4.1. Animals Study Design and Ethics Statement

The initial aim of this study was to explore the recovery mechanism of diabetes-induced ED through Ago2 protein (Mybiosource Inc., San Diego, CA, USA) treatment. Animal care and experimental procedures followed the Animal Care and Use Subcommittee of Inha University (INHA 220831-839). In this study, a total of 70 eight-week-old C57BL/6J mice were used (Orient Bio, Inc., Seongnam, Republic of Korea). Type 1 diabetes was induced as previously described [53] through intraperitoneal injections of multiple low streptozotocin doses (STZ, 50 mg/kg body weight in 0.1 M citrate buffer, pH 4.5, Sigma-Aldrich, St. Louis, MO, USA) for 5 consecutive days. To test the efficacy of Ago2, STZ-induced type-1 diabetic mice received two intracavernous injections of PBS (Gibco, Carlsbad, CA, USA) or Ago2 proteins (−3 and 0; 1, 5, and 10 μg in 20 μL of PBS). After 2 weeks, we evaluated erectile function in mice through electrical stimulation of the cavernous nerve. We performed this experiment twice for Western blot and histological examination studies. Fasting and postprandial blood glucose levels were measured 8 weeks after STZ injection using an Accu-Check blood glucose meter (Roche Diagnostics, Mannheim, Germany). Mean systolic blood pressure (MSBP) was evaluated by a noninvasive tail-cuff system (Visitech System, Apex, NC, USA). All experiments in this study were performed blindly, and no mice died during these experiments.

### 4.2. Cell Culture

Primary mouse endothelial cells (MCECs) were prepared and maintained as previously described [54]. The penis tissue was harvested, then the glans, urethra, and dorsal neurovascular bundle were removed; only the cavernous tissue was placed into a sterilized tube containing Hank’s balanced salt solution (HBSS, Gibco, Carlsbad, CA, USA). Cavernous tissues were cut into approximately 1~2 mm fragments, put into 60 mm dishes, and covered by Matrigel (Becton Dickinson, Mountain View, CA, USA). Tissues were cultured with the complement M199 medium (Gibco, Carlsbad, CA, USA) containing 20% fetal bovine serum (FBS, Gibco, Carlsbad, CA, USA), 0.5 mg/mL of heparin (Sigma-Aldrich, St. Louis, MO, USA), 5 ng/mL of recombinant human vascular endothelial growth factor (VEGF, R&D Systems Inc., Minneapolis, MN, USA), and 1% penicillin/streptomycin (Gibco, Carlsbad, CA, USA) in a 5% CO2 atmosphere incubator at 37 °C. After cells were confluent on the bottom of the 60 mm cell culture dishes (approximately two weeks of culture), sprouting cells were sub-cultured into other cell culture dishes coated with 0.2% gelatin (Sigma-Aldrich, St. Louis, MO, USA). Cells from passages 2 to 4 were available for all experiments. Human umbilical vein endothelial cells (HUVECs, Lonza, Cohasset, MN, USA) were cultured according to the ATCC guidelines. All HUVECs between passages 2 and 7 were used in this study.

### 4.3. In Vitro Tube Formation Assay

MCECs and HUVECs were used for tube formation assays as previously described [38]. Cells were briefly cultured in complement medium M199 (Gibco, Carlsbad, CA, USA) under normal-glucose (5 mM, Sigma-Aldrich, St. Louis, MO, USA) or high-glucose (30 mM) conditions for at least 3 days, with or without Ago2 proteins (100, 200, and 500 ng/mL of cultured medium). Tube formation assays were performed in a 48-well plate with 100 μL of growth factor-reduced Matrigel (Becton Dickinson, Mountain View, CA, USA). Phase images of the tube formation were observed and taken by a phase-contrast microscope (CKX41, Olympus, Tokyo, Japan) at 18 h, and master junctions were blindly counted using ImageJ software ver 1.52q17 (National Institutes of Health [NIH], Bethesda, MD, USA).

### 4.4. Cell Migration Assay

The SPL ScarTMBlock system (SPL Life Sciences, Pocheon-si, Gyeonggi-do, Republic of Korea) was used to create uniform scratches for cell migration. MCECs and HUVECs were seeded into the block system at >90% confluence in 60 mm culture dishes, as previously described [55]. After 5 h, blocks were removed, and the cells were further incubated in a cultured medium with 2% FBS and thymidine (2 mM, Sigma-Aldrich, St. Louis, MO, USA) for 24 h. Images were taken with a phase-contrast microscope (Olympus, Tokyo, Japan). Migrated cells were blindly analyzed by measuring the ratio of cells that moved into the frame line shown in Figure 2 using ImageJ software ver 1.52q17 (National Institutes of Health [NIH], USA).

### 4.5. Measurement of Erectile Function

Mouse erectile function was evaluated by the Biopac Student Lab System (Biopac Systems Inc., Goleta, CA, USA), as previously described [43]. The parameters were set as follows: the voltage was 5 V, frequency 12 Hz, pulse width 1 ms, and duration 1 min. Mice were anesthetized with xylazine (5 mg/kg, Bayer Korea, Seoul, Republic of Korea) and ketamine (100 mg/kg, Yuhan Corp., Seoul, Republic of Korea). The cavernous nerve was dissected and exposed. Next, a bipolar platinum wire electrode was placed around the cavernous nerve and stimulated with the setting value. The maximum intracavernous pressure (ICP) and total ICP (total ICP was defined as the area under the curve from cavernous nerve stimulation initiation until 20 s after stimulus termination) were recorded. Blood pressure variations between individuals were normalized by the ratios of maximal ICP and total ICP to MSBP.

### 4.6. TUNEL Assay

Frozen tissue sections and cells were stained following the manufacturer’s instructions for an ApopTag Fluorescein In Situ Apoptosis Detection Kit (Chemicon, Temecula, CA, USA). Samples were then stained with the endothelial cell marker CD31 (1:50; Millipore, Temecula, CA, USA) and the corresponding secondary antibody. At the last stage, a mounting solution containing 4,6-diamidino-2-phenylindole (DAPI; Vector Laboratories, Inc., Burlingame, CA, USA) was used to mount the slide and stain nuclei. Digital images at a magnification of 100× were obtained with a confocal fluorescence microscope (K1-Fluo; Nanoscope Systems, Inc., Daejeon, Republic of Korea). The number of apoptotic cells was counted in a blind manner using ImageJ software ver 1.52q17 (National Institutes of Health [NIH], USA).

### 4.7. Histologic Examinations

The 12 μm-thick frozen tissue sections were incubated with the corresponding primary antibody against Ago2 (1:100, Abcam, Cambridge, MA, USA), CD31 (1:50; Millipore, Temecula, CA, USA), NG2 (1:50; Millipore, Temecula, CA, USA), neurofilament (1:50; Sigma-Aldrich, St. Louis, MO, USA), neuronal nitric oxide synthase (nNOS, 1:50; Santa Cruz, Dallas, TX, USA), phosphohistone H3 (1:50; Millipore, Temecula, CA, USA), or nitrotyrosine (1:50; Millipore, Temecula, CA, USA) at 4 °C overnight as previously described [5]. After several PBS washes (Gibco, Carlsbad, CA, USA), sections were incubated with Fluorescein (FITC) -conjugated goat anti-Armenian hamster IgG (1:200; Jackson ImmunoResearch Laboratories, West Grove, PA, USA), donkey anti-rabbit Dylight 550 (1:200; Abcam, Cambridge, MA, USA), Rhodamin (TRITC)-conjugated AffiniPure rabbit anti-mouse IgG (1:200; Jackson ImmunoResearch Laboratories, West Grove, PA, USA), donkey anti-mouse Alexa Fluor 488 (1:200; Jackson ImmunoResearch Laboratories, West Grove, PA, USA), or secondary antibodies for 2 h at room temperature. Sections were mounted in a solution containing 4,6-diamidino-2-phenylindole (DAPI; Vector Laboratories, Inc., Burlingame, CA, USA) for nuclei staining. Signals were visualized using a confocal microscope (K1-Fluo, Nanoscope Systems, Inc., Daejeon, Republic of Korea), and sample quantification was blindly analyzed using ImageJ software ver 1.52q17 (National Institutes of Health [NIH], USA).

### 4.8. In Situ Detection of Superoxide Anion

For superoxide anion detection, we used hydroethidine (Molecular Probes, Eugene, OR, USA), an oxidative fluorescent dye, to detect intracellular superoxide anions in situ, as previously described [53]. Frozen tissue sections were briefly stained with the immunofluorescent CD31 (1:50; Millipore, Temecula, CA, USA) primary antibody and corresponding second antibody. Before mounting, tissue sections were incubated with hydroethidine (1:10,000; Molecular Probes, Eugene, OR, USA) for 30 min at room temperature, avoiding light. Finally, tissue sections were mounted in a solution containing DAPI (Vector Laboratories, Inc., Burlingame, CA, USA) for nuclei staining, and fluorescent signals were visualized using a confocal microscope (K1-Fluo, Nanoscope Systems, Inc., Daejeon, Republic of Korea). Fluorescence-positive expression of ethidium bromide in endothelial cells was blindly quantified using ImageJ software ver 1.52q17 (National Institutes of Health [NIH], USA).

### 4.9. Western Blot 

For the immunoblot analyses, equal protein amounts (30 μg/lane) were electrophoresed on sodium dodecyl sulfate-polyacrylamide gels (4% to 20%) and transferred to polyvinylidene fluoride membranes. After blocking with 5% nonfat dry milk at room temperature for 1 h, the membranes were probed with primary antibodies as follows: Ago2 (1:1000, Abcam, Cambridge, MA, USA), eNOS (1:1000; BD, Mountain View, CA, USA), p-eNOS Ser^1177^ (1:1000; Cell Signaling, Beverly, MA, USA), NF-κB (1:1000; Cell Signaling, Beverly, MA, USA), p-NF-κB Ser^536^ (1:1000; Invitrogen, Carlsbad, CA, USA), and β-actin (1:5000; Santa Cruz, Dallas, TX, USA). The signals were visualized using the ECL (Amersham Pharmacia Biotech, Piscataway, NJ, USA) detection system. The Western blot band densitometry images were quantified using ImageJ software ver 1.52q17 (National Institutes of Health [NIH], USA).

### 4.10. Statistical Analysis

Data are expressed as means ± SEMs of at least four independent experiments. The unpaired *t*-test was used to compare two groups, and a one-way ANOVA followed by Tukey’s post hoc test was used for four group comparisons. The analysis was conducted using GraphPad Prism version 8 (GraphPad Software Inc., San Diego, CA, USA), and statistical significance was accepted for *p* values < 0.05.

## 5. Conclusions

In summary, our results indicated that Ago2 expression is high in neurovascular tissues and significantly decreased under diabetic conditions. Exogenous delivery of Ago2 protein improves neurovascular regeneration by restoring cavernous endothelial cell and neuronal cell content, reducing ROS productions, and inducing eNOS/NF-κB signaling, ultimately improving erectile function in diabetic mice. Our study provides new insights into Ago2 function and may represent a new therapeutic approach for ED and other vascular and neurological diseases.

## Data Availability

The data that support the findings of this study are available from the corresponding author upon reasonable request.

## Supplementary Materials

The following supporting information can be downloaded at: https://www.mdpi.com/article/10.3390/ijms24032935/s1.

## References

[1] Kolluru G.K., Bir S.C., Kevil C.G. (2012). Endothelial dysfunction and diabetes: Effects on angiogenesis, vascular remodeling, and wound healing. Int. J. Vasc. Med..

[2] Gur S., Peak T.C., Kadowitz P.J., Sikka S.C., Hellstrom W.J. (2014). Review of erectile dysfunction in diabetic animal models. Curr. Diabetes Rev..

[3] Angulo J., Gonzalez-Corrochano R., Cuevas P., Fernandez A., La Fuente J.M., Rolo F., Allona A., Saenz de Tejada I. (2010). Diabetes exacerbates the functional deficiency of NO/cGMP pathway associated with erectile dysfunction in human corpus cavernosum and penile arteries. J. Sex Med..

[4] Cayetano-Alcaraz A.A., Tharakan T., Chen R., Sofikitis N., Minhas S. (2022). The management of erectile dysfunction in men with diabetes mellitus unresponsive to phosphodiesterase type 5 inhibitors. Andrology.

[5] Yin G.N., Choi M.J., Kim W.J., Kwon M.H., Song K.M., Park J.M., Das N.D., Kwon K.D., Batbold D., Oh G.T. (2014). Inhibition of Ninjurin 1 restores erectile function through dual angiogenic and neurotrophic effects in the diabetic mouse. Proc. Natl. Acad. Sci. USA.

[6] Bennett N.E., Kim J.H., Wolfe D.P., Sasaki K., Yoshimura N., Goins W.F., Huang S., Nelson J.B., de Groat W.C., Glorioso J.C. (2005). Improvement in erectile dysfunction after neurotrophic factor gene therapy in diabetic rats. J. Urol..

[7] Burchardt M., Burchardt T., Anastasiadis A.G., Buttyan R., de la Taille A., Shabsigh A., Frank J., Shabsigh R. (2005). Application of angiogenic factors for therapy of erectile dysfunction: Protein and DNA transfer of VEGF 165 into the rat penis. Urology.

[8] Hu L., Qi S., Zhang K., Fu Q. (2018). Essential role of brain-derived neurotrophic factor (BDNF) in diabetic erectile dysfunction. Andrologia.

[9] Israeli J.M., Lokeshwar S.D., Efimenko I.V., Masterson T.A., Ramasamy R. (2022). The potential of platelet-rich plasma injections and stem cell therapy for penile rejuvenation. Int. J. Impot. Res..

[10] Manfredi C., Castiglione F., Fode M., Lew-Starowicz M., Romero-Otero J., Bettocchi C., Corona G., ESSM Scientific Collaboration and Partnership (ESCAP) (2022). News and future perspectives of non-surgical treatments for erectile dysfunction. Int. J. Impot. Res..

[11] Yin G.N., Jin H.R., Choi M.J., Limanjaya A., Ghatak K., Minh N.N., Ock J., Kwon M.H., Song K.M., Park H.J. (2018). Pericyte-Derived Dickkopf2 Regenerates Damaged Penile Neurovasculature Through an Angiopoietin-1-Tie2 Pathway. Diabetes.

[12] Liu J., Carmell M.A., Rivas F.V., Marsden C.G., Thomson J.M., Song J.J., Hammond S.M., Joshua-Tor L., Hannon G.J. (2004). Argonaute2 is the catalytic engine of mammalian RNAi. Science.

[13] Arroyo J.D., Chevillet J.R., Kroh E.M., Ruf I.K., Pritchard C.C., Gibson D.F., Mitchell P.S., Bennett C.F., Pogosova-Agadjanyan E.L., Stirewalt D.L. (2011). Argonaute2 complexes carry a population of circulating microRNAs independent of vesicles in human plasma. Proc. Natl. Acad. Sci. USA.

[14] Jagannathan R., Thapa D., Nichols C.E., Shepherd D.L., Stricker J.C., Croston T.L., Baseler W.A., Lewis S.E., Martinez I., Hollander J.M. (2015). Translational Regulation of the Mitochondrial Genome Following Redistribution of Mitochondrial MicroRNA in the Diabetic Heart. Circ. Cardiovasc. Genet..

[15] Pozzi A., Dowling D.K. (2022). New Insights into Mitochondrial-Nuclear Interactions Revealed through Analysis of Small RNAs. Genome Biol. Evol..

[16] Zhang X., Zuo X., Yang B., Li Z., Xue Y., Zhou Y., Huang J., Zhao X., Zhou J., Yan Y. (2014). MicroRNA directly enhances mitochondrial translation during muscle differentiation. Cell.

[17] Ye Z.L., Huang Y., Li L.F., Zhu H.L., Gao H.X., Liu H., Lv S.Q., Xu Z.H., Zheng L.N., Liu T. (2015). Argonaute 2 promotes angiogenesis via the PTEN/VEGF signaling pathway in human hepatocellular carcinoma. Acta Pharmacol. Sin..

[18] Machado-Pereira M., Saraiva C., Bernardino L., Cristovao A.C., Ferreira R. (2022). Argonaute-2 protects the neurovascular unit from damage caused by systemic inflammation. J. Neuroinflamm..

[19] Tattikota S.G., Rathjen T., McAnulty S.J., Wessels H.H., Akerman I., van de Bunt M., Hausser J., Esguerra J.L., Musahl A., Pandey A.K. (2014). Argonaute2 mediates compensatory expansion of the pancreatic beta cell. Cell Metab..

[20] Iosue I., Quaranta R., Masciarelli S., Fontemaggi G., Batassa E.M., Bertolami C., Ottone T., Divona M., Salvatori B., Padula F. (2013). Argonaute 2 sustains the gene expression program driving human monocytic differentiation of acute myeloid leukemia cells. Cell Death Dis..

[21] Neppl R.L., Kataoka M., Wang D.Z. (2014). Crystallin-alphaB regulates skeletal muscle homeostasis via modulation of argonaute2 activity. J. Biol. Chem..

[22] Kim B.S., Jung J.S., Jang J.H., Kang K.S., Kang S.K. (2011). Nuclear Argonaute 2 regulates adipose tissue-derived stem cell survival through direct control of miR10b and selenoprotein N1 expression. Aging Cell.

[23] Florijn B.W., Duijs J., Levels J.H., Dallinga-Thie G.M., Wang Y., Boing A.N., Yuana Y., Stam W., Limpens R., Au Y.W. (2019). Diabetic Nephropathy Alters the Distribution of Circulating Angiogenic MicroRNAs Among Extracellular Vesicles, HDL, and Ago-2. Diabetes.

[24] Wu S., Yu W., Qu X., Wang R., Xu J., Zhang Q., Xu J., Li J., Chen L. (2014). Argonaute 2 promotes myeloma angiogenesis via microRNA dysregulation. J. Hematol. Oncol..

[25] Cheng N., Li Y., Han Z.G. (2013). Argonaute2 promotes tumor metastasis by way of up-regulating focal adhesion kinase expression in hepatocellular carcinoma. Hepatology.

[26] Naoghare P.K., Tak Y.K., Kim M.J., Han E., Song J.M. (2011). Knock-down of argonaute 2 (AGO2) induces apoptosis in myeloid leukaemia cells and inhibits siRNA-mediated silencing of transfected oncogenes in HEK-293 cells. Basic Clin. Pharmacol. Toxicol..

[27] Volpe C.M.O., Villar-Delfino P.H., Dos Anjos P.M.F., Nogueira-Machado J.A. (2018). Cellular death, reactive oxygen species (ROS) and diabetic complications. Cell Death Dis..

[28] Carmell M.A., Xuan Z., Zhang M.Q., Hannon G.J. (2002). The Argonaute family: Tentacles that reach into RNAi, developmental control, stem cell maintenance, and tumorigenesis. Genes Dev..

[29] Cerutti L., Mian N., Bateman A. (2000). Domains in gene silencing and cell differentiation proteins: The novel PAZ domain and redefinition of the Piwi domain. Trends Biochem. Sci..

[30] Muller M., Fazi F., Ciaudo C. (2019). Argonaute Proteins: From Structure to Function in Development and Pathological Cell Fate Determination. Front. Cell Dev. Biol..

[31] Li J., Kim T., Nutiu R., Ray D., Hughes T.R., Zhang Z. (2014). Identifying mRNA sequence elements for target recognition by human Argonaute proteins. Genome Res..

[32] Cefalu W.T. (2006). Animal models of type 2 diabetes: Clinical presentation and pathophysiological relevance to the human condition. ILAR J..

[33] Anita L., Yin G.N., Hong S.S., Kang J.H., Gho Y.S., Suh J.K., Ryu J.K. (2022). Pericyte-derived extracellular vesicle-mimetic nanovesicles ameliorate erectile dysfunction via lipocalin 2 in diabetic mice. Int. J. Biol. Sci..

[34] Choi J.S.Y., de Haan J.B., Sharma A. (2022). Animal models of diabetes-associated vascular diseases: An update on available models and experimental analysis. Br. J. Pharmacol..

[35] Ghatak K., Yin G.N., Hong S.S., Kang J.H., Suh J.K., Ryu J.K. (2022). Heat Shock Protein 70 in Penile Neurovascular Regeneration Requires Cystathionine Gamma-Lyase. World J. Mens. Health.

[36] Parikh J., Zemljic-Harpf A., Fu J., Giamouridis D., Hsieh T.C., Kassan A., Murthy K.S., Bhargava V., Patel H.H., Rajasekaran M.R. (2017). Altered Penile Caveolin Expression in Diabetes: Potential Role in Erectile Dysfunction. J. Sex Med..

[37] Purvis G.S.D., Chiazza F., Chen J., Azevedo-Loiola R., Martin L., Kusters D.H.M., Reutelingsperger C., Fountoulakis N., Gnudi L., Yaqoob M.M. (2018). Annexin A1 attenuates microvascular complications through restoration of Akt signalling in a murine model of type 1 diabetes. Diabetologia.

[38] Yin G.N., Kim D.K., Kang J.I., Im Y., Lee D.S., Han A.R., Ock J., Choi M.J., Kwon M.H., Limanjaya A. (2022). Latrophilin-2 is a novel receptor of LRG1 that rescues vascular and neurological abnormalities and restores diabetic erectile function. Exp. Mol. Med..

[39] Bobbie M.W., Roy S., Trudeau K., Munger S.J., Simon A.M., Roy S. (2010). Reduced connexin 43 expression and its effect on the development of vascular lesions in retinas of diabetic mice. Investig. Ophthalmol. Vis. Sci..

[40] Eftekhari A., Vahed S.Z., Kavetskyy T., Rameshrad M., Jafari S., Chodari L., Hosseiniyan S.M., Derakhshankhah H., Ahmadian E., Ardalan M. (2020). Cell junction proteins: Crossing the glomerular filtration barrier in diabetic nephropathy. Int. J. Biol. Macromol..

[41] Li A.F., Roy S. (2009). High glucose-induced downregulation of connexin 43 expression promotes apoptosis in microvascular endothelial cells. Investig. Ophthalmol. Vis. Sci..

[42] Tien T., Barrette K.F., Chronopoulos A., Roy S. (2013). Effects of high glucose-induced Cx43 downregulation on occludin and ZO-1 expression and tight junction barrier function in retinal endothelial cells. Investig. Ophthalmol. Vis. Sci..

[43] Jin H.R., Kim W.J., Song J.S., Piao S., Choi M.J., Tumurbaatar M., Shin S.H., Yin G.N., Koh G.Y., Ryu J.K. (2011). Intracavernous delivery of a designed angiopoietin-1 variant rescues erectile function by enhancing endothelial regeneration in the streptozotocin-induced diabetic mouse. Diabetes.

[44] Musicki B., Kramer M.F., Becker R.E., Burnett A.L. (2005). Inactivation of phosphorylated endothelial nitric oxide synthase (Ser-1177) by O-GlcNAc in diabetes-associated erectile dysfunction. Proc. Natl. Acad. Sci. USA.

[45] Griendling K.K., Sorescu D., Ushio-Fukai M. (2000). NAD(P)H oxidase: Role in cardiovascular biology and disease. Circ. Res..

[46] Luczak K., Balcerczyk A., Soszynski M., Bartosz G. (2004). Low concentration of oxidant and nitric oxide donors stimulate proliferation of human endothelial cells in vitro. Cell Biol. Int..

[47] Stone J.R., Collins T. (2002). The role of hydrogen peroxide in endothelial proliferative responses. Endothelium.

[48] Morgan M.J., Liu Z.G. (2011). Crosstalk of reactive oxygen species and NF-kappaB signaling. Cell Res..

[49] Noort A.R., van Zoest K.P., Weijers E.M., Koolwijk P., Maracle C.X., Novack D.V., Siemerink M.J., Schlingemann R.O., Tak P.P., Tas S.W. (2014). NF-kappaB-inducing kinase is a key regulator of inflammation-induced and tumour-associated angiogenesis. J. Pathol..

[50] Taniguchi K., Karin M. (2018). NF-kappaB, inflammation, immunity and cancer: Coming of age. Nat. Rev. Immunol..

[51] Jeremy J.Y., Jones R.A., Koupparis A.J., Hotston M., Persad R., Angelini G.D., Shukla N. (2007). Reactive oxygen species and erectile dysfunction: Possible role of NADPH oxidase. Int. J. Impot. Res..

[52] Tirichen H., Yaigoub H., Xu W., Wu C., Li R., Li Y. (2021). Mitochondrial Reactive Oxygen Species and Their Contribution in Chronic Kidney Disease Progression Through Oxidative Stress. Front. Physiol..

[53] Yin G.N., Shin T.Y., Ock J., Choi M.J., Limanjaya A., Kwon M.H., Liu F.Y., Hong S.S., Kang J.H., Gho Y.S. (2022). Pericyte-derived extracellular vesicles-mimetic nanovesicles improves peripheral nerve regeneration in mouse models of sciatic nerve transection. Int. J. Mol. Med..

[54] Yin G.N., Ryu J.K., Kwon M.H., Shin S.H., Jin H.R., Song K.M., Choi M.J., Kang D.Y., Kim W.J., Suh J.K. (2012). Matrigel-based sprouting endothelial cell culture system from mouse corpus cavernosum is potentially useful for the study of endothelial and erectile dysfunction related to high-glucose exposure. J. Sex Med..

[55] Yin G.N. (2022). Pericyte-derived heme-binding protein 1 promotes angiogenesis and improves erectile function in diabetic mice. Investig. Clin. Urol..

