# Supplementary material for: Argonaute 2 Restores Erectile Function by Enhancing Angiogenesis and Reducing Reactive Oxygen Species Production in Streptozotocin (STZ)-Induced Type-1 Diabetic Mice
Source: Int J Mol Sci. 2023 Feb 2;24(3):2935. doi: 10.3390/ijms24032935 (PMC9918048; doi:10.3390/ijms24032935)
Supplement: Supplementary file 1 [file ijms-24-02935-s001.zip › ijms-2159427-SI.pdf]

Supplemental Information

Argonaute 2 restores erectile function by enhancing angiogenesis and reducing reactive oxygen species production in streptozotocin (STZ)-induced type 1 diabetic mice

Fang-Yuan Liu, Guo Nan Yin, Jiyeon Ock, Fitri Rahma Fridayana, Lashkari Niloofar, Yan Huang, Minh Nhat Vo, Jun-Kyu Suh, Soon-Sun Hong, Ju-Hee Kang, and Ji-Kan Ryu

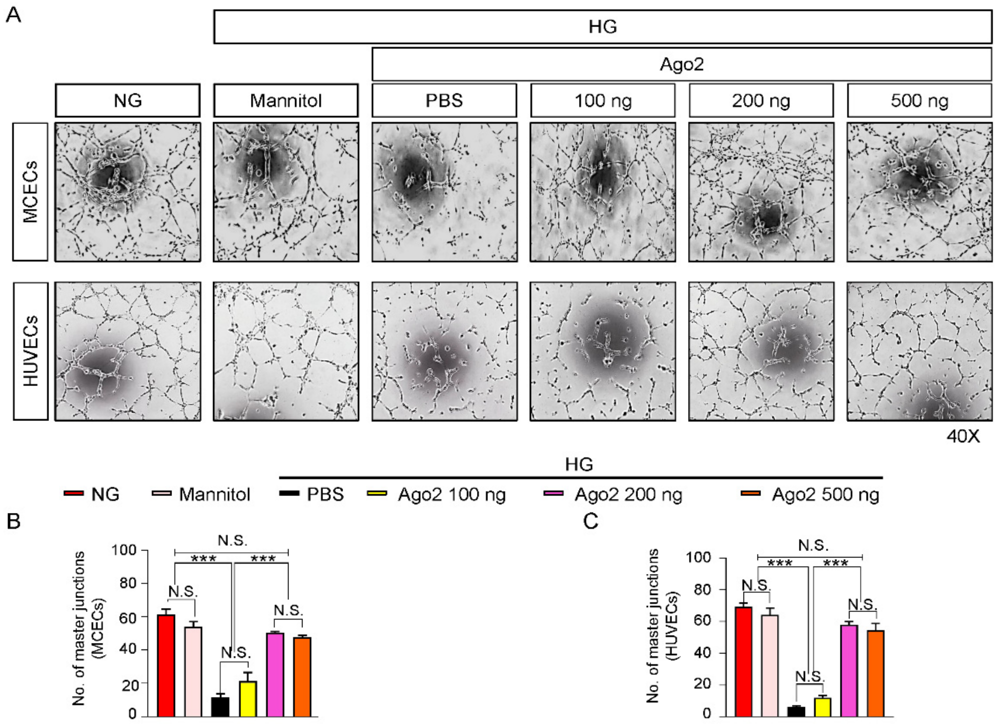

Figure S1. Determination of Ago2 protein optimal dosage to induce angiogenesis in endothelial cells under high-glucose conditions. (**A**) Tube formation assays in MCECs (**A,** top) and HUVECs (**A,** bottom) exposed to NG, mannitol (an osmotic control), or HG with PBS or Ago2 proteins (100 ng/mL, 200 ng/mL, and 500 ng/mL, respectively). (**B** and **C**) The number of master junctions in MCECs (**B**) and HUVECs (**C**) were quantified using ImageJ software (n = 4, ***P< 0.001). Magnification, 40 ×. The results were presented as mean ± SEMs. MCECs, mouse cavernous endothelial cells; HUVECs, Human umbilical vein endothelial cells; NG, normal-glucose; HG, high-glucose; N.S., not significant.

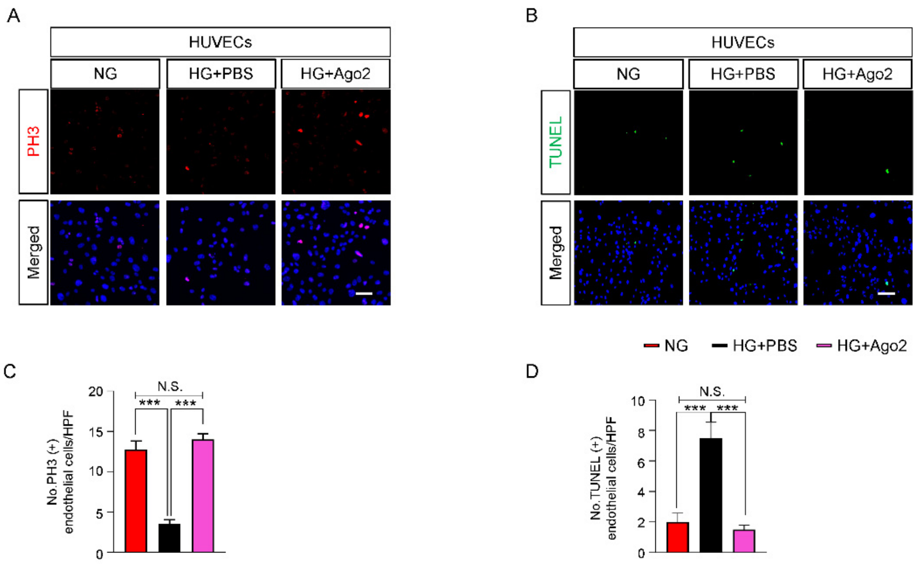

Figure S2. Ago2 induces HUVEC proliferation and reduces apoptosis under high glucose conditions. (**A** and **B**) Immunofluorescence staining for PH3 (red, **A**) and TUNEL (green, **B**) in HUVECs exposed to NG, or HG with PBS or Ago2 proteins (200 ng/mL), respectively. Scale bars, 50 μm. Nuclei were labeled with DAPI (blue). (**C** and **D**) The number of PH3-positive (**C**) or TUNEL-positive (**D**) endothelial cells was quantified using ImageJ software (n = 4, ***P< 0.001). The results were presented as mean ± SEMs. HUVECs, Human umbilical vein endothelial cells; DAPI, 4,6-diamidino-2-phenylindole; NG, normal-glucose; HG, high-glucose; N.S., not significant.

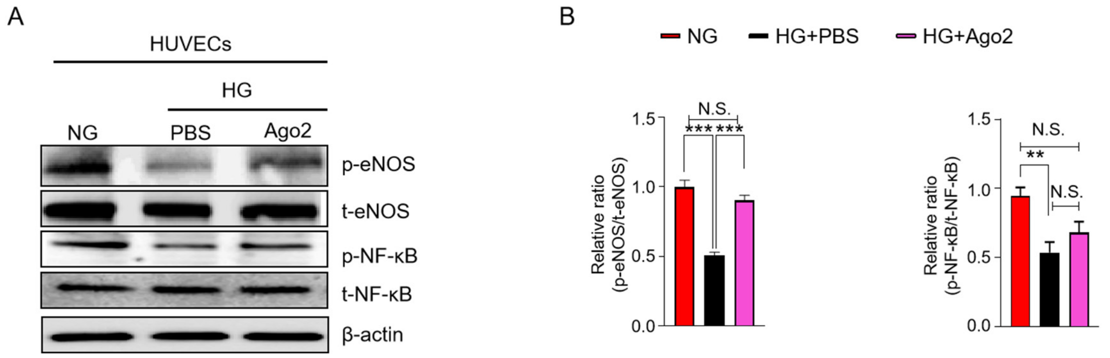

Figure S3. Ago2 induces eNOS Ser^{1177} phosphorylation in HUVECs under high-glucose conditions. (**A**) Representative western blots for p-eNOS Ser^{1177}, total eNOS,

p-NF-κB Ser^{536}, and total NF-κB in HUVECs exposed to NG, or HG with PBS or Ago2 protein (200 ng/mL). (**B** and **C**) Normalized band intensity values were quantified using ImageJ software (n = 4, **P< 0.01, ***P< 0.001). The results were presented as mean ± SEMs. The value expressed as ratios of the NG group was arbitrarily set to 1. HUVECs, Human umbilical vein endothelial cells; NG, normal-glucose; HG, high-glucose; N.S., not significant.

Table S1. Physiologic and metabolic parameters: 2 weeks after treatment with Ago2

		STZ-induced diabetic mice			
	Control	PBS	1 μg Ago2	5 μg Ago2	20 μg Ago2
Body weight (g)	32.7±1.3	32.6±1.1*	22.4±1.2*	22.3±1.1*	22.5±1.4*
Fasting glucose (mg/dl)	101.4±4.8	472.0±31.3*	481±26.8*	482.6±27.1*	488.0±56.0*
Postprandial glucose (mg/dl)	170.4±8.0	569±8.5*	563±7.4*	588±8.9*	576.2±16.8*
MSBP (mm Hg)	98.3±4.4	100.8±4.4	98.7±3.5	98.1±3.0	97.4±3.9

Values are the mean±SEM for n=10 for each group. *$P<0.05$ vs. Control group; STZ, streptozotocin; MSBP, mean systolic blood pressure.
